# Soil Bacterial Diversity and Its Relationship with Soil CO_2_ and Mineral Composition: A Case Study of the Laiwu Experimental Site

**DOI:** 10.3390/ijerph17165699

**Published:** 2020-08-07

**Authors:** Hongying Zhang, Zongjun Gao, Mengjie Shi, Shaoyan Fang

**Affiliations:** College of Earth Science and Engineering, Shandong University of Science and Technology, Qingdao 266590, China; zhy10sdust@126.com (H.Z.); smj_sdust@126.com (M.S.); fsysdust@126.com (S.F.)

**Keywords:** soil bacteria, soil CO_2_, land use type, mineral composition, Alpha diversity

## Abstract

To better understand the characteristics of soil bacterial diversity in different environments, the Laiwu Qilongwan experimental site was selected as it is of great significance for the study of geochemical cycles. The soil CO_2_, mineral composition and bacterial community were analyzed by an EGM-4 portable environmental gas detector, an X-ray diffractometer and 16S rDNA high-throughput sequencing, and soil bacterial diversity and the relationship between soil bacterial diversity and environmental factors were studied. The results showed that with increasing soil depth, the CO_2_ content increased, the feldspar and amphibole contents increased, the quartz content decreased, the richness of the soil bacterial community increased, the relative richness of Nitrospirae increased, and Chloroflexi decreased. The dominant bacteria were Proteobacteria, Actinobacteria and Acidobacteria. There were slight differences in soil CO_2_, mineral composition and dominant bacterial flora at the same depth. Actinobacteria, Proteobacteria and Firmicutes were the dominant phyla of L02. The CO_2_ was lowest in bare land, and the quartz and K-feldspar contents were the highest. Soil CO_2_ mainly affected the deep bacterial diversity, while shallow soil bacteria were mainly affected by mineral components (quartz and K-feldspar). At the same depth, amphibole and clay minerals had obvious effects on the bacterial community, while CO_2_ had obvious effects on subdominant bacteria.

## 1. Introduction

The soil sphere is an integral part of the Earth system. Soil microorganisms are the most active part of the soil and the main component of the soil decomposition system. Soil microorganisms play an important role in soil formation and development, organic matter decomposition, material transformation, energy transfer, the geochemical cycle and bioremediation [1]. Due to the transitional exploitation of natural resources, biodiversity has been destroyed, and ecosystems have exhibited fluctuations. As a sensitive indicator for stabilizing ecosystems and monitoring soil quality changes, soil microbial diversity plays an important role in evaluating ecosystems and maintaining ecological balance. An increasing number of scholars have focused on the research and protection of soil microbial diversity, the analysis of diversity characteristics and the impact of environmental factors on diversity.

Soil microbial diversity is a newly emerging interdisciplinary subject that has recently attracted increased attention in the fields of soil science, microbiology and biodiversity. Studies on soil microbial diversity are of great significance for exploring natural biological mechanisms, coping with global climate change, controlling various kinds of environmental pollution, maintaining ecological service functions and promoting the sustainable use of soil. Many factors affect the soil microbial diversity and structure, especially its functional diversity, and these factors can be roughly divided into natural and human factors. Natural factors include agricultural vegetation type [2,3], soil type [4], temperature and moisture [5]; human factors include pesticides, fertilization [6] and soil tillage methods [7]. Leguminous plants can significantly increase the number of microorganisms, microbial biomass and community metabolic diversity [2]. A previous researcher found that the total number of soil microorganisms under different vegetation cover changed as follows: grassland > crop cover > bare land, without fertilizer input for a long time [6]. Waldrop et al. found that soil organic carbon decreased by 50–55% and microbial biomass decreased by 75% under the conversion of forest to arable land [8]. The simulation results showed that continuous warming significantly increased the soil microbial biomass C and N contents in the Chongming Dongtan wetland, and the microbial biomass increased by 39.32%, 70.79%, 65.20%, and 74.09% from the surface layer to the deep layer (0–10, 10–20, 20–30, and 30–40 cm, respectively) [9]. Biodiversity is closely related to the structure and function of the whole ecosystem. Biodiversity is the main component required to maintain soil productivity and one of the important indicators for evaluating soil quality [10]. Microorganisms live in soil; thus, the species, quantity and dynamic distribution of microorganisms exhibit various differences in response to the influence of soil conditions, plant communities and climate conditions. Early studies found that long-term fertilization of soil resulted in increased microbial biomass and microbial activity [11]. Pang et al. found that the soil microbial activity in farmland and orchards with field management measures was generally higher than that in grassland soil [12]. An increase in plant diversity can provide more types of food for soil microorganisms, which is conducive to the improvement of microbial diversity [13]. Previous studies have shown that the response of soil microorganisms to the external environment is affected by the resources available in soil [14]. Soil pH is another important factor affecting microorganisms, and pH has a significant impact on the structure of the microbial community and the soil metabolic process in which microorganisms participate [15]. Tiedje J. M proposed that the composition of the soil microbial community was greatly influenced by vegetation types [16]. They believed that there was a positive correlation between soil microbial diversity and plant community diversity [17]. Johnson et al. applied the intergenic transcribed spacer (ITS) method to study the relationship between soil microbial communities and crop species in San Joaquin Valley, USA. This study found that soil microbial species in different soils planted with tomatoes and grapes were very similar, while the soil microbial communities in different soils planted with cotton or safflower were very different [18].

Soil is the largest organic carbon pool in terrestrial ecosystems. Soil not only provides a carbon source for vegetation growth and maintains the good physical structure of soil but also releases carbon to the atmosphere, such as CO_2_. Because of the large storage capacity of soil carbon, a small variation in soil carbon can lead to a large fluctuation in atmospheric CO_2_, which plays an extremely important role in the global carbon cycle [19,20]. The dynamic balance of soil carbon not only directly affects soil fertility and crop yield but is also an important index to be considered in soil quality assessments and sustainable land use management. Carbon sequestration and emissions also have an important impact on the content of greenhouse gases in the atmosphere and global climate change. Soil carbon is affected by many physical, biological and human factors, such as climate, vegetation, physical and chemical soil characteristics and human activities, and the interaction between these factors is especially important, which is crucial to the dynamic changes in soil carbon [21,22,23]. Soil has become an important priority area in the study of the carbon cycle and global change in terrestrial ecosystems [24,25,26]. The soil carbon cycle and soil microbial community were found to respond to elevated atmospheric CO_2_ concentrations [27]. High CO_2_ concentrations not only promote the growth of the plant root system but also increase the amount of plant root secretions and change the quality and quantity of root secretions and plant residue litter. These changes indirectly affect the composition, structure, activity and function of soil microbial communities, which use these substances as nutrients and energy sources and subsequently affect the process of soil carbon deposition and the carbon cycle [28]. Studies on poplar and herbaceous plants found that fungal abundance changed significantly under high CO_2_ concentrations [29]. However, it was found that CO_2_ concentration could increase fungal abundance in paddy soil [30]. Under high CO_2_ concentrations, the community structure characteristics of microorganisms change significantly. The proportion of photosynthetic carbon in the underground part and soil carbon storage was also found to change with changes in microbial activities. Studies on soil microorganisms should aim at a combination of macrogenomics and soil ecology using both high-throughput sequencing and gene chip technology. The results of such studies would not only clearly indicate important geochemical processes of the carbon and nitrogen cycle in the soil, which are a key function of microbial populations, but also help to more comprehensively and accurately reveal the environmental factors impacting the microbial community, such as the influence of carbon dioxide on the soil rhizosphere microbial community. At the same time, clear metagenomics should be used to reveal important geochemical processes of the carbon and nitrogen cycle, which are key functions of microbial populations in response to environmental factor changes. Such studies are of great significance for the systematic analysis of microbial gene functional diversity and the discussion of the effect of CO_2_ on important microbial functional populations and soil carbon storage.

Soil minerals are the remnants and newly formed minerals of bedrock weathering and parent materials through soil formation. Soil minerals are the main components of solid soil particles, including various primary minerals, layered silicate clay minerals and oxides of iron, aluminum and manganese. Approximately 80–90% of the microorganisms living in soil adhere to the surface of minerals or mineral–organic complexes to form a single microbial colony or biofilm [31]. The results showed that the adsorption capacity of cadmium on soil colloids and minerals (especially kaolin and goethite) was significantly improved by adding bacteria [32]. Brown et al. studied the relationship between biofilm and mineral transformation and found that oxides and hydroxides can migrate and dissolve magnetite particles in gray granite and transform them into hematite during the interactions among bacteria [33]. The 16S rDNA high-throughput sequencing results indicated that the variation in the microbial community was correlated with the concentration of released As (R = 0.7, Pb < 0.05), and iron-reducing bacteria, including Pseudomonas, Clostridium and Geobacter, were the main drivers of As mobilization from the sediments at depths of 26 m and 36 m [34]. Few studies have examined soil microorganism–mineral interactions in situ; however, Certini et al. (2004) reported a microbial community structure in rock fragments that differed from the surrounding soil [35].

There are many factors affecting the diversity of the soil microbial community structure, including the soil physical and chemical properties and the vegetation community types, such as soil particle size, water content, pH, enzyme activity and organic matter content, which directly affect the quantity and activity of soil microorganisms. In summary, previous studies on soil microbial diversity under different conditions have mainly focused on soil properties and human factors. Few studies on the impact of soil CO_2_ changes on microbial diversity have been conducted. To analyze the influence of soil CO_2_ and mineral composition on the microbial community, soil microorganisms in different environments were selected for analysis in this study. The relationship between the microbial community and environmental factors was illustrated through analysis of carbon dioxide and mineral composition. The results of this study have certain guiding significance for the future study of microbial action during the process of the geochemical carbon cycle.

## 2. Materials and Methods

### 2.1. Study Area

**Figure 1:**
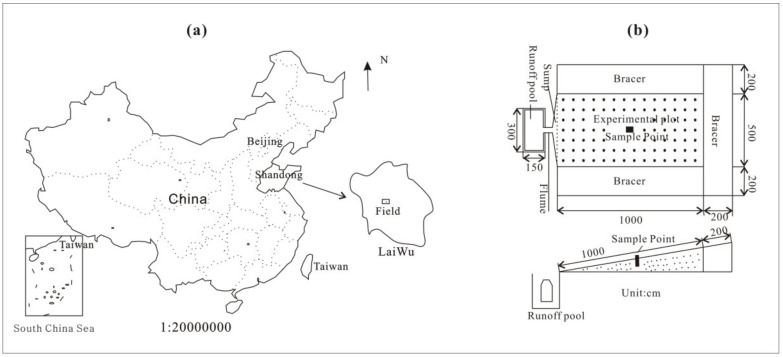
Diagram of experimental plot location (**a**) is a map of the location of the experimental plot. (**b**) is the experimental plot.

The park that was used in this study is located in Qilongwan village of Laiwu (Figure 1) at 117°38′ E, 36°22′ N. The park belongs to the hilly area located in the middle and south of Shandong Province, and it represents the main type of soil erosion in the northern mountainous area. The climate is temperate humid and semihumid continental monsoon. The climate in this region is characterized by obvious seasonal differences: cold with little snow in winter, dry and windy in spring, hot and rainy in summer, and dry and cool in autumn. In 2012, the park was named the “National Water and Soil Conservation Science and Technology Demonstration Park” by the Ministry of Water Resources. The soil layer in the experimental plot is brown soil. The depth is approximately 70–80 cm; the soil is light brown sand with a slightly granular structure, and it is loose and porous. The plant roots are developed, and animal caverns exist. The bedrock is mainly granite and gneiss. The experimental plot was designed to be 5 m wide, 10 m long and 50 m^2^ in the horizontal projection area, and the angle of the experimental plot was 15°. Prefabricated bracers were arranged along the upper part and both sides of the plot to establish a protection zone with a width of 2 m (Figure 1). According to the layout of the test site, five test plots were selected for monitoring and sampling (Figure 1). The samples are shown in Table 1. To analyze the soil bacterial diversity under different conditions, two conditions (depth and vegetation) were set up to analyze the soil bacterial diversity. To determine the influence of depth on soil bacterial diversity, bare land was selected, and 0–15 cm, 30–45 cm, and 60–75 cm mixed soil samples were collected for testing (W01–W03). To observe the effect of vegetation on soil bacterial diversity, four vegetation types (peanut, peach, chestnut, pine) were selected for observation at the experimental site (L02–L05), and bare land was used as the control group (L01 and W01), with a sampling depth of 0–15 cm.

### 2.2. Methods

#### 2.2.1. Soil Sampling and Particle Size Analysis

The soil samples were collected using the diagonal sampling method, and the soil samples taken at depths of 0–15 cm, 30–45 cm, and 60–75 cm at the four diagonals and the center were selected for mixing. The soil from all the sampling points was mixed together, and the roots, rocks and weeds were removed from the soil. Eight soil samples were collected, each weighing 250 g. The samples were divided into three parts, and particle size analysis, mineral analysis and microbial analysis were carried out. The soil samples were dried at an oven temperature of 100 °C for 5 h to a constant weight for particle size analysis. According to the size and distribution range of the tested samples, sieves with different sizes and holes were stacked together for screening, the sieve allowance of each sieve was collected, and the particle size distribution of the tested samples by weight was obtained. The ranges of granule grading were selected as 20 mm, 10 mm, 5 mm, 1 mm, 0.5 mm, 0.25 mm, 0.1 mm and 0.075 mm. After screening, the weight was measured and calculated, and finally, the granule grading curve was obtained.

#### 2.2.2. The Method for Soil CO_2_ and Mineral Composition

The soil CO_2_ flux measurement system (an EGM-4 portable environmental gas detector, PP Systems, 110 Haverhill Road, Suite 301 Amesbury, MA 01913, USA) was used to detect the soil CO_2_ concentration. The test was conducted at depths of 0–15 cm, 30–45 cm, and 60–75 cm, according to the thickness of the soil layer. To avoid interference from other factors, the test points were selected uniformly. The soil CO_2_ test time was 9:00–11:00 a.m. because a previous study found that the soil CO_2_ content during this period could represent the average value for the whole day [36]. The soil CO_2_ content in the soil was measured by hammering a 2 cm diameter iron drill in the vertical direction at the test point. Starting from the surface, the soil CO_2_ content was measured at a depth of 15 cm. After drilling to the specified depth, the iron rod was pulled out, and a rubber hose connected to the inlet of the EGM-4 portable environmental gas detector was quickly inserted into the borehole. The maximum soil CO_2_ concentration readings were measured by the instrument after a short delay.

To understand the main mineral composition of the study area, the mineral composition was analyzed by X-ray diffraction (XRD) [37]. The X-ray diffractometer used in this study was the X’PertPRO produced by Panaco Company in the Netherlands. The parameters for the instrument were as follows: pipe pressure 40 kV, pipe flow 40 mA, soller slit 2.5°, divergent slit 1.0 mm, divergent slit hole angle 1°, anti-scattering slit 1.0 mm, and accept slit 0.2 m. The step scanning method was adopted, the scanning step was 0.02 degrees, the scanning speed was 0.02°·s^−1^, and the scanning range was 5–70°. After completing the qualitative analysis, jEdit was combined with TOPAS-Academic software to carry out the refinement of Rietveld full spectrum fitting. For samples, the refinement process was based on the samples of all phases of the crystallographic information file (CIF) structure. Point-by-point comparisons of the diffraction intensity value and observation value, which were calculated by the least squares method, were conducted to obtain minerals by adjusting the peak shape parameters and structure parameters to quantitative data, including the background, crystal cell parameters, atomic placeholder (occ), temperature factor (beq), and preferred orientation of possible correction, to ensure that the calculated peak shape and experimental peak shape reached the greatest extent. Finally, MDI Jade6.0 software was used to determine the mineral composition and content according to the test energy spectrum curve.

#### 2.2.3. Soil Bacterial Test and Analysis Method

First, approximately 10–20 g of sample was placed in refrigerated storage at 4 °C for soil bacterial detection. The specific test methods and data processing were as follows:

(1) DNA extraction and PCR amplification

**Figure 2:**
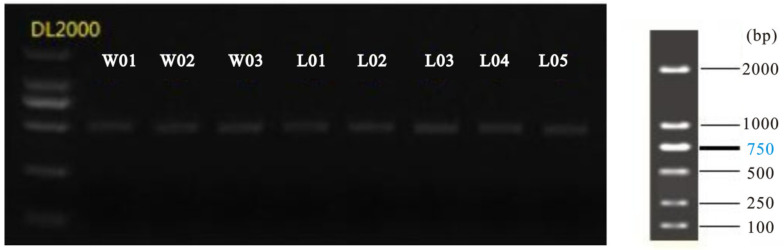
The results of PCR amplification were identified by 2% agarose gel.

Microbial community genomic DNA was extracted from soil samples using the E.Z.N.A.^®^ soil DNA Kit (Omega Bio-tek, Norcross, GA, USA) according to the manufacturer’s instructions. The DNA extract was checked on 1% agarose gel, and DNA concentration and purity were determined with a NanoDrop 2000 UV-vis spectrophotometer (Thermo Scientific, Wilmington, NC, USA). The hypervariable regions V4–V5 of the bacterial 16S rRNA gene were amplified with primer pairs 515F (5′-GTGCCAGCMGCCGCGG-3′) and 907R (5′-CCGTCAATTCMTTTRAGTTT-3′) by an ABI GeneAmp^®^ 9700 PCR thermocycler (ABI, CA, USA). The PCR amplification of 16S rRNA gene was performed as follows: initial denaturation at 95 °C for 3 min, followed by 27 cycles of denaturing at 95 °C for 30 s, annealing at 55 °C for 30 s and extension at 72 °C for 45 s, single extension at 72 °C for 10 min, and end at 4 °C. The PCR mixtures contain 5× TransStart FastPfu buffer 4 μL, 2.5 mM dNTPs 2 μL, forward primer (5 μM) 0.8 μL, reverse primer (5 μM) 0.8 μL, TransStart FastPfu DNA Polymerase 0.4 μL, template DNA 10 ng, and finally ddH_2_O up to 20 μL. PCR reactions were performed in triplicate. The PCR product was extracted from 2% agarose gel and purified using the AxyPrep DNA Gel Extraction Kit (Axygen Biosciences, Union City, CA, USA) according to the manufacturer’s instructions and quantified using a Quantus™ Fluorometer (Promega, Madison City, WI, USA) (Figure 2).

(2) Illumina MiSeq sequencing

Purified amplicons were pooled in equimolar and paired-end sequenced on an Illumina MiSeq PE300 platform/NovaSeq PE250 platform (Illumina, San Diego, CA, USA) according to the standard protocols by Majorbio Bio-Pharm Technology Co. Ltd. (Shanghai, China). The raw reads were deposited into the NCBI Sequence Read Archive (SRA) database.

(3) Processing of sequencing data

The raw 16S rRNA gene sequencing reads were demultiplexed, quality-filtered by fastp version 0.20.0 and merged by FLASH version 1.2.7 with the following criteria: (i) the 300 bp reads were truncated at any site receiving an average quality score of <20 over a 50 bp sliding window, and the truncated reads shorter than 50 bp were discarded, reads containing ambiguous characters were also discarded; (ii) only overlapping sequences longer than 10 bp were assembled according to their overlapped sequence. The maximum mismatch ratio of overlap region was 0.2. Reads that could not be assembled were discarded; (iii) Samples were distinguished according to the barcode and primers, and the sequence direction was adjusted, with exact barcode matching, and 2 nucleotide mismatches in the primer matching. Operational taxonomic units (OTUs) with 97% similarity cutoff were clustered using UPARSE version 7.1, and chimeric sequences were identified and removed. The taxonomy of each OTU representative sequence was analyzed by Ribosomal Database Project (RDP) Classifier version 2.2 against the 16S rRNA database using a confidence threshold of 0.7 (www.i-sanger.com) [38].

The bacterial diversity and abundance of different bacteria in the samples were based on the analysis of OTUs. To obtain the corresponding species classification information for each OTU, the RDP classifier Bayesian algorithm was used to classify and analyze the OTU representative sequences at a 97% similarity level, and the community composition of each sample was counted at each classification level: domain, kingdom, phylum, class, order, family, genus and species. Venn graphs were used to calculate the number of common and unique species (such as OTUs) in multiple samples to intuitively reflect the similarity and overlap of species in environmental samples. The Alpha diversity analysis (including the Chao1 index, ACE value, Shannon index and Simpson index [39]) was used to indicate the community structure and diversity abundance of bacteria in the samples.

By analyzing the bacterial richness in samples and according to 97% similarity, the sequence was clustered into OTUs by using UCLUST software, the RDP classification was selected to annotate the sequence, and the taxonomic map of the bacterial phylum was obtained. Two aspects of information can be presented intuitively: the bacteria contained in each sample at a taxonomic level and the relative abundance of each bacteria in the sample [40,41]. Principal component analysis (PCoA) is a nonconstrained data dimensionality reduction analysis method. The soil bacterial communities of three vertical and five horizontal samples were analyzed by using PCoA (OTU level) with QIIME software. Species-sample data (97% similarity sample OTU table) were used to perform detrended correspondence analysis (DCA), which provided the lengths of the gradient along the first axis in the analysis results. If the length is greater than 4.0, canonical correspondence analysis (CCA) should be selected; if it is between 3.0 and 4.0, both redundancy analysis (RDA) and CCA can be selected; if it is less than 3.0, the result of RDA is better than that of CCA. DCA was performed by using CANOCO 4.5 software under different conditions. The results showed that the maximum eigenvalues of the four axes were 1.30 and less than 3.0 [42].

## 3. Results

### 3.1. Soil CO_2_

**Figure 3:**
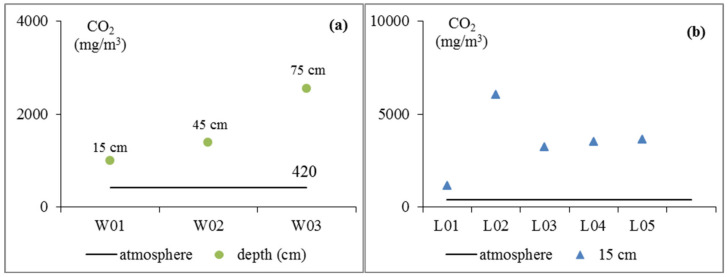
The change in soil CO_2_ concentrations (the horizontal line shows the amount of CO_2_ in the atmosphere. (**a**) shows the soil CO_2_ concentration at different depths, and (**b**) shows the soil CO_2_ concentration at 15 cm under different vegetation types).

The content of atmospheric CO_2_ was 420 mg/m^3^. Figure 3 shows that the soil CO_2_ at each point was different and larger than the amount of atmospheric CO_2_. Figure 3a shows that the soil CO_2_ concentration increased with depth. Earlier, relevant experts and scholars discovered similar patterns [43,44,45]; for example, an increase in soil CO_2_ concentration in the aerated zone with depth was discovered at the Amagosa Desert Research Institute of the United States Geological Survey [46]. Some studies found that soil CO_2_ concentration showed a two-way gradient trend with depth [47], whereas some studies found that soil CO_2_ decreased with depth [48]. Figure 3b shows that there are some differences in soil CO_2_ at 15 cm depth. The soil CO_2_ content was the lowest in L01 at only 960 mg/m^3^. The soil CO_2_ content was the highest in L02. At the same depth, the soil CO_2_ concentrations with different land uses were different [49].

### 3.2. Mineral Composition

**Figure 4:**
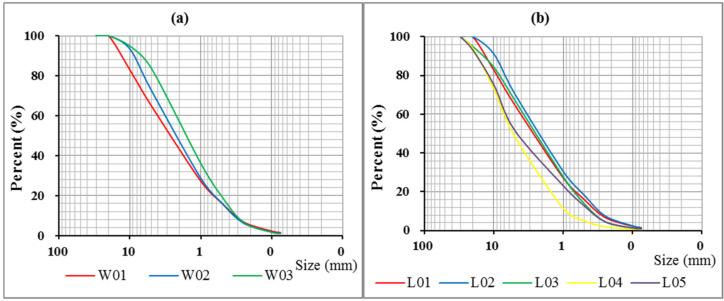
Grain size analysis curves. (**a**) shows the soil size at different depths, and (**b**) shows the soil size at 15 cm under different vegetation types).

The soil samples were screened, and particle size distribution curves were drawn (Figure 4). The graph of particle size distribution reflects the relative content of particles in soil samples. According to the gradient of the particle size distribution curve, the uniformity or gradation of particles in soil samples can be roughly judged. A steep curve indicates that the particle sizes are similar and the particles are uniform; a gentle curve indicates that the particle sizes differ greatly, the soil particles are not uniform and the gradation is good. The curves of W01, W02 and W03 were basically similar, indicating that the distribution of particles in longitudinal soil samples was relatively uniform. The curves of L01, L02 and L03 were basically similar in the transverse direction, but there was a gap between L04 and L05.

The results of the XRD mineral composition analysis are shown in Table 2. The main minerals in each sample were quartz, K-feldspar and plagioclase. The distribution of calcite content in each soil sample was basically the same. Clay minerals represented 10.4% in L04 and L05. The amphibole content in L01 was very small. In summary, the mineral compositions of soil samples in both the horizontal and vertical directions show that soil minerals were mainly silicate minerals, while carbonate minerals contain only a small amount of calcite, and there were some differences among the mineral compositions. There were dozens of primary minerals in the soil, including quartz, feldspar, mica, amphibole, and pyroxene [50]. There was also a certain correlation between soil mineral composition and particle size. The smaller the particle size is, the higher the mass fraction of primary minerals and the lower the mass fraction of secondary minerals [51,52]. The mass fractions of primary minerals in W01 (L01), W02 and W03 were 91%, 91.3% and 89.2%, respectively. The contents of primary minerals in L02–L05 were 90.2%, 89.2%, 86.4%, and 86.7%. In this study, it was found that the main minerals were quartz and feldspar, and the feldspar content was significantly higher than the quartz content; however, some scholars found that the soil mineral composition in semiarid areas is mainly quartz, and the contents of amphibole and feldspar are relatively low [51,53]. In this study, the content of quartz was higher in the surface soil and decreased with increasing depth, which is similar to previous research results [51,54].

### 3.3. Soil Bacterial Diversity

#### 3.3.1. Rarefaction Curve of the Soil Bacterial Community

**Figure 5:**
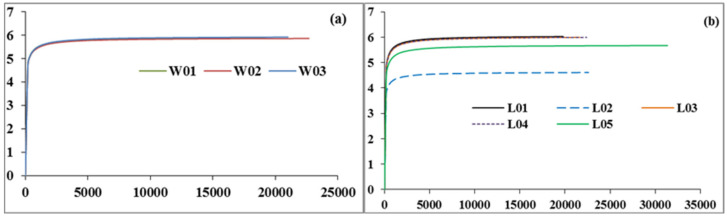
Rarefaction curve of the soil bacterial community ((**a**) is the soil bacterial rarefaction curve at different depths, and (**b**) is the rarefaction curve at 15 cm under different vegetation types).

In microbial diversity analysis, it is necessary to verify whether the amount of sequencing data is sufficient to reflect the species diversity in samples, and the dilution curve can be used to test this index. The dilution curve is used to calculate the expected number of OTUs when extracting n reads (where n is less than the total number of reads) by using the relative proportion of the known OTUs in the 16S rDNA sequence; then, a curve can be created according to the expected number of OTUs corresponding to a set of N values (generally a set of sequences with equal differences less than the total number of reads). When the curve tends to flatten or reaches the plateau stage, it can be considered that the depth of sequencing has basically covered all species in the sample; in contrast, it means that the species diversity in the sample is high, and there are many species not detected by sequencing. As shown in Figure 5, the curves of different samples tend to be flat, indicating that the samples selected in this experiment can cover all kinds of microorganisms, indicating the diversity of microorganisms in each sample.

#### 3.3.2. Venn Diagram Analysis of Species

**Figure 6:**
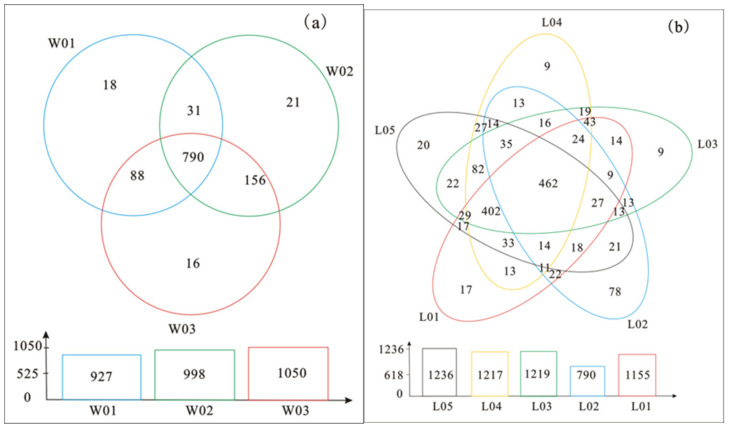
The Venn diagrams of samples ((**a**) shows the number of operational taxonomic units (OTUs) of soil bacteria at different depths, and (**b**) shows the number of OTUs at 15 cm under different vegetation types).

Venn diagrams can be used to count the number of common and unique species (such as OTUs) in multiple groups or multiple samples. These diagrams can intuitively show the similarity and overlap of species in environmental samples. As shown in Figure 6a, the number of core OTUs was 790, and the number of unique OTUs was 55 in the three groups of samples. There were 927 OTUs in W01, and there were 18 unique OTUs; there were 998 OTUs in total and 16 unique OTUs in W02; there were 1050 OTUs in total and 21 unique OTUs in W03. The data showed that the W01, W02 and W03 microbial species exhibited individuality and commonness, and their commonness was greater than their individuality. There were obvious differences between the W02 microbial species and the other two samples, and the similarity between the W01 and W03 microbial species was high. Under the same conditions, soil microorganisms at different depths had obvious commonness, and there were a few specific microorganisms. The microbial species increased with increasing depth. Figure 6b shows that there were obvious differences in the classification of microbial species at the same depth and under different conditions. This result indicates that the common microorganisms of L01, L03, L04 and L05 were obvious, and there were distinct microbial species in L02. There were obvious commonalities among the microbial species at different depths and distinct individualities among microbial species at the same depths. It was concluded that both depth and external conditions can affect the species of microorganisms, but external conditions had a good influence on the formation of new species of microorganisms.

#### 3.3.3. Abundance of Bacterial Communities

Alpha diversity analysis can indicate the community structure and diversity abundance of microorganisms, including the Chao1 index, ACE value, Shannon index and Simpson index [39]. Sobs represent the number of bacterial species observed in each sample. The number of OTUs in a community is estimated by the Chao1 algorithm. Chao1 is often used to estimate the total number of species in ecology and was first proposed by Chao (1984). The larger the Chao1 value is, the greater the total number of species. ACE is one of the most commonly used indices for estimating the total number of species in ecology, which is different from the Chao1 index. The higher the value of Chao1 or ACE is, the higher the richness of the community. One of the indices used to estimate bacterial diversity in samples, as proposed by Edward Hugh Simpson (1949), is the Simpson index and is often used in ecology to quantitatively describe the biodiversity of a region. The Shannon index can also be used to estimate bacterial diversity in samples. The higher the Shannon index or the lower the Simpson index is, the higher the diversity of the bacterial community.

Table 3 shows that there were some differences in the indices of the samples. From W01, W02 and W03, the Shannon index increased slightly, while the Simpson index decreased to some extent, indicating that community diversity decreased, and bacterial diversity at W01 was the highest. The Chao index and ACE index increased, indicating that bacterial richness was highest at W03, while that at W01 was the lowest. The Shannon index and Simpson index of L01 were the highest, and the Simpson index was the lowest, which indicated that the diversity of the bacterial community in L01 was high. The smallest Shannon index and the largest Simpson index in L02 indicated a low diversity of the bacterial community. The Shannon index and Simpson index of L03 and L04 were basically the same, indicating that the diversity of bacterial communities had some similarities. The Chao index and ACE index of L01 were the lowest, indicating that the bacterial richness was the lowest. The Chao index and ACE index of L03 were the largest, indicating that the bacterial richness was the highest. The bacterial richness of other samples was second.

#### 3.3.4. OTU-Level Bacterial Diversity Analysis

**Figure 7:**
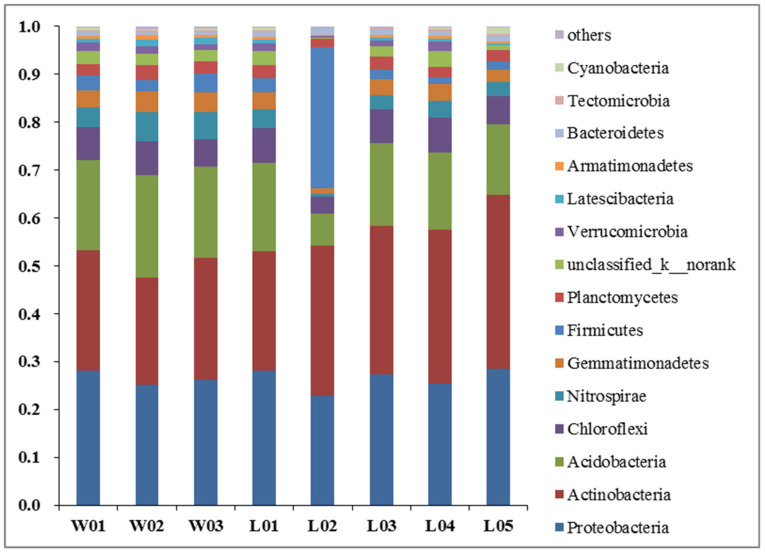
Distribution of relative abundance of different bacterial community structures at phylum levels of different treatments in soil (W01–W03 represents soil microorganisms of each sample vertically, and L01–L05 represents soil microorganisms of each sample horizontally).

By analyzing the microbial richness in samples and according to 97% similarity, the sequences were clustered into OTUs by using UCLUST software, the RDP classification was selected to annotate the sequences, and the taxonomic map of the microbial phylum was obtained [40,41]. According to the analysis results (Figure 7), 15 bacterial taxa, 16 bacterial classes and 33 genera were detected vertically (W01–W03); 15 bacterial taxa, 19 bacterial classes and 41 genera were detected horizontally (L01–L05), and bacteria with a relative abundance less than 0.01 were merged into other groups. As shown in Figure 7, the vertical soil microbial species mainly originate from nine main categories, of which Proteobacteria, Actinobacteria, and Acidobacteria were dominant and Chloroflexi, Nitrospirae, Gemmatimonadetes, Firmicutes, and Planctomycetes were subdominant. The relative abundances of Proteobacteria in each sample (W01–W03) were 0.28, 0.25 and 0.26, the relative abundances of Actinobacteria in each sample were 0.25, 0.22 and 0.26, and the relative abundances of Acidobacteria in each sample were 0.18, 0.21 and 0.19, respectively. The relative abundances of Chloroflexi, Nitrospirae, Gemmatimonadetes, Firmicutes and Planctomycetes in W01 were 0.07, 0.04, 0.03, 0.03 and 0.02, respectively, and those in W02 were 0.07, 0.06, 0.04, 0.02 and 0.03, respectively. The relative abundances of Chloroflexi, Nitrospirae, Gemmatimonadetes, Firmicutes and Planctomycetes in W03 were 0.06, 0.06, 0.04, 0.04 and 0.02, respectively. The Proteobacteria content was the highest in W01 and W02, while the Actinobacteria content in W03 was slightly higher than the Proteobacteria content. With increasing depth, the content of Proteobacteria in the soil decreased slightly, and the content of subdominant bacteria increased to a certain extent. In addition, Deinococcus-Thermus and Chloroflexi with relative abundances less than 0.01 existed in only W01, while trace Chlamydiae appeared in W03. These results showed that depth can change the content of microbial species and reduce or increase some special microflora present in small contents. There were two main factors that could be considered to explain the obvious difference in L02. One factor was that one week before the soil sample was collected and tested, fertilization treatment was carried out to promote the growth of the peanut crop, which led to significant changes in microbial species [7,55]; the other was that shallow peanut roots were relatively developed and had different root respiration effects on soil microorganisms [56]. L01 was bare land, and L03, L04 and L05 were planted with perennial woody plants, which do not require annual fertilization; thus, the distribution of soil microorganisms was relatively stable in these plots. The results show that land use at the same depth can significantly change the dominant and subdominant microflora of soil microorganisms and can also reduce or increase the special microflora.

## 4. Discussion

### 4.1. PCoA

**Figure 8:**
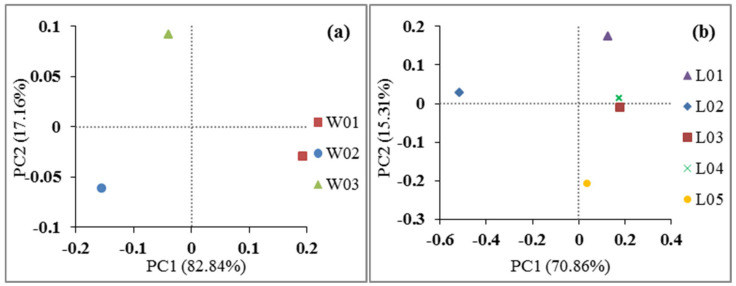
Principal component analysis (PCoA) of soil bacterial community diversity. (**a**) represents the samples from different depths, and (**b**) represents the samples from different vegetation types.

Figure 8a shows the results of PCoA, which indicates that the interpretation rates of the ranking results by two axes were 82.84% and 17.16%. W01 is in the lower right part of the graph, W02 is in the lower left part of the graph, and W03 is in the upper part of the graph. Figure 8b shows that the interpretation rates of the ranking results from the two axes were 70.86% and 15.31%. L02 is on the left side of the graph, while other samples are clustered on the right side of the graph. L03 and L04 were similar. The results showed that there were significant differences in bacterial communities in the vertical direction, but the largest differences were found in L02 and other bacterial communities in the horizontal direction. This finding is similar to the results of bacterial community analysis.

### 4.2. RDA

**Figure 9:**
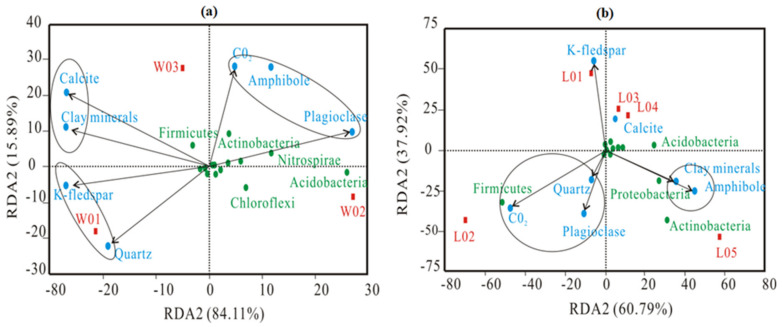
Redundancy analysis (RDA) of the bacterial community species (phylum) in soil and the environmental factors; (**a**) represents the samples from different depths, and (**b**) represents the samples from different vegetation types. (The red rectangles are the sample groups under different environments; the green circles are the bacterial community species (phylum); the blue circles are the environmental factors. The length of the arrow of environmental factors represents the degree of the influence (explanatory quantity) of environmental factors on species data; the angle between the arrows of environmental factors represents a positive and negative correlation (acute angle: positive correlation; obtuse angle: negative correlation; right angle: no correlation); the arrow represents the projection from the sample point to the environmental factors, and the distance from the projection point to the origin represents the distance between the environmental factors and the sample, which represents the relative influence of the community distribution).

The RDA results showed that there was a certain correlation between environmental factors and bacterial communities, as shown in Figure 9. Figure 9a shows that quartz and K-feldspar were the main factors influencing the bacterial community distribution in W01. Plagioclase was the main factor influencing the bacterial community distribution in W02, while soil CO_2_ and amphibole were the main factors influencing the bacterial community distribution in W03, indicating that the environmental factors affecting bacterial community distribution at different depths were different. Soil CO_2_, clay minerals and calcite had a great influence on Firmicutes distribution, while Actinobacteria distribution was mainly affected by soil CO_2_ and amphibole. Plagioclase affected the Acidobacteria, Nitrospirae and Gemmatimonadetes distribution. The correlation between environmental factors and bacterial community distribution shown is Figure 9b is significantly different from that shown in Figure 9a. K-feldspar was the main environmental factor influencing the L01 bacterial community distribution, soil CO_2_ was the main environmental factor influencing the L02 bacterial community distribution, calcite was the main environmental factor influencing the L03 and L04 bacterial community distribution, while amphibole and clay minerals were the main environmental factors influencing the L05 bacterial community distribution; these results indicated that the environmental factors affecting bacterial community distribution under different conditions may be the same. The distribution of Firmicutes was mainly affected by soil CO_2_. Calcite and clay minerals can affect the distribution of Acidobacteria. Actinobacteria and Proteobacteria were mainly affected by amphibole and clay minerals. Soil CO_2_ was positively correlated with Firmicutes, and amphibole was positively correlated with Actinobacteria. Other mineral components had different effects on the bacterial community distribution under different conditions. It was found that soil CO_2_ and amphibole were the main environmental factors affecting bacterial community distribution.

### 4.3. Correlation Heatmap Analysis

**Figure 10:**
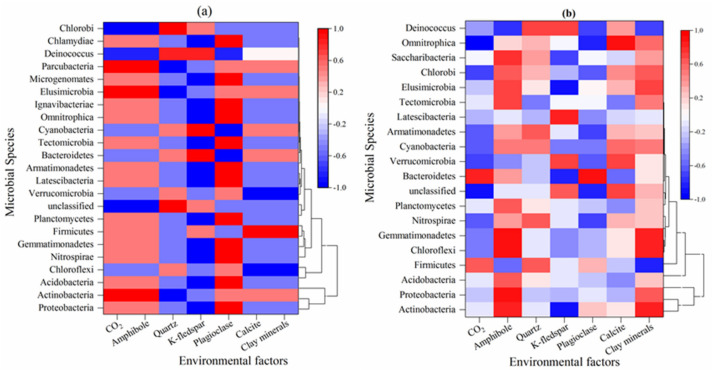
Heatmap of Spearman’s correlation coefficients between bacterial community species (phylum) in soil and environmental factors ((**a**) is the vertical heatmap of W01–W03, and (**b**) is the horizontal heatmap of L01–L05).

To further clarify the relationship between the bacterial community and environmental factors, Spearman correlation analysis was carried out for the different taxa of bacteria and environmental factors. The results showed that (Figure 10a) Proteobacteria, Actinobacteria, Acidobacteria, Nitrospirae, Gemmatimonadetes, Firmicutes, and Planctomycetes were positively correlated with CO_2_ and amphibole, and CO_2_ and amphibole were negatively correlated with Chloroflexi. Proteobacteria, Acidobacteria, Chloroflexi, Nitrospirae, Gemmatimonadetes, and Planctomycetes were negatively correlated with calcite and clay minerals, while calcite and clay minerals were positively correlated with Actinobacteria and Firmicutes. Quartz and K-feldspar were negatively correlated with the dominant phyla (Proteobacteria, Actinobacteria, and Acidobacteria). Quartz was positively correlated with the subdominant phylum Chloroflexi. K-feldspar was positively correlated with Firmicutes. The effects of plagioclase and K-feldspar on bacterial flora communities were significantly opposite. Simultaneously, it can be seen that the effects of CO_2_ and amphibole on bacterial communities are common and positively correlated with most bacterial communities. Calcite and clay minerals also had some common effects on the bacterial communities but were negatively correlated with most bacterial communities, while the effects of quartz and K-feldspar on bacterial communities were slightly different. The results showed that environmental factors had some common effects on the bacterial community at different depths (CO_2_ and amphibole, calcite and clay minerals), and there were also significant differences (plagioclase and K-feldspar). Figure 10b shows that the correlation between the bacterial community and environmental factors was obviously different. Actinobacteria, Proteobacteria, and Acidobacteria were slightly negatively correlated with CO_2_. Chloroflexi, Gemmatimonadetes, and Nitrospirae were significantly negatively correlated with CO_2_, while Firmicutes was significantly positively correlated with CO_2_. Amphibole was positively correlated with dominant phyla (Actinobacteria, Proteobacteria, and Acidobacteria) and subdominant bacteria (Chloroflexi, Gemmatimonadetes, Nitrospirae, and Planctomycetes) but negatively correlated with Firmicutes. The correlations between quartz and Actinobacteria, Proteobacteria, Acidobacteria, Chloroflexi, Gemmatimonadetes, and Nitrospirae were general, but there was a significant positive correlation between quartz and Firmicutes. K-feldspar was negatively correlated with dominant and subdominant bacteria, of which Actinobacteria was the most correlated. The correlation between dominant and subdominant phyla and plagioclase was general, but plagioclase was negatively correlated with Nitrospirae. Actinobacteria, Proteobacteria, Acidobacteria, Chloroflexi, Gemmatimonadetes, Nitrospirae, Planctomycetes and clay minerals were positively correlated, while Firmicutes and clay minerals were negatively correlated. Calcite was generally associated with bacterial communities. The results showed that different environmental factors had different effects on the diversity distribution of bacterial communities. Amphibole and clay minerals had the greatest impact on the diversity of dominant bacterial communities, while other environmental factors had a slightly lower impact on dominant bacterial communities. CO_2_ had a great impact on the diversity of bacterial subdominant flora but a small impact on dominant flora. This result was significantly different from that in Figure 10a, mainly because soil CO_2_ increased with depth. The results showed that soil CO_2_, amphibole, quartz, K-feldspar, plagioclase, calcite, and clay minerals could affect the bacterial community composition to varying degrees. Amphibole and clay minerals were significantly correlated with dominant bacteria (Actinobacteria, Proteobacteria, and Acidobacteria) under different external conditions, while soil CO_2_ was inversely correlated with dominant bacteria. K-feldspar could significantly affect Actinobacteria and Proteobacteria. It also showed that soil CO_2_ and amphibole had the greatest impact on bacterial community composition under different conditions, which fully confirmed that carbon dioxide played an important role in the process of bacterial diversity research.

Soil minerals are the remnants and newly formed minerals of bedrock weathering and parent materials through soil formation. Soil minerals are the main components of solid soil particles, including various primary minerals, layered silicate clay minerals and oxides of iron, aluminum and manganese. Approximately 80–90% of the bacteria living in soil adhere to the surface of minerals or mineral–organic complexes to form a single bacterial colony or biofilm [31]. Brown et al. studied the relationship between biofilm and mineral transformation and found that oxides and hydroxides can migrate and dissolve magnetite particles in gray granite and transform the particles into hematite during their interactions with different bacteria [33]. The 16S rRNA high-throughput sequencing results indicated that the variation in the bacterial community was correlated with the released concentration, and iron-reducing bacteria, including Pseudomonas, Clostridium and Geobacter, were the main drivers of As mobilization from the sediments at depths of 26 m and 36 m [34]. The interaction between minerals and bacteria is a common basic biogeochemical process in nature [57], such as the element cycle and water chemical composition. Minerals provide nutrients for the life activities of bacteria, and the metabolic activities of bacteria can also change the solubility of minerals [58,59]. Early bacterial mineral mechanisms were widely used in heavy metal pollution control [60,61]; for example, clay minerals can react with surrounding redox bacteria, resulting in significant changes in soil physical and chemical properties [61], and there was a significant positive correlation between clay minerals and soil bacterial biomass [62]. It was found that bacteria can increase the solubility of plagioclase by producing organic acids under laboratory culture conditions [63,64]. Under the same soil layer thickness, there was a significant difference between carbonate development and the total number of soil bacteria [65]. In this paper, at different depths, Actinobacteria, Proteobacteria, Acidobacteria, and clay minerals were positively correlated. At present, there is no consistent understanding of how CO_2_ affects bacterial diversity [66,67,68]. Some researchers found that CO_2_ had a significant impact on bacterial community diversity, such as Feng [69] and Dunbar [70]. It has also been found that CO_2_ has no effect on bacterial diversity, such as in Drigo [71] and Guenet [67].

## 5. Conclusions

In this paper, the changes in soil bacterial community structure and diversity were analyzed by collecting soil samples under different vegetation and soil depth conditions. The main conclusions are as follows:

There were obvious differences in CO_2_ content and mineral composition in soils under different environments. The CO_2_ content increases with depth. At the same depth, the content of CO_2_ in the soil was also different, and bare land had the lowest CO_2_ content. With increasing soil depth, the content of quartz decreased, the contents of K-feldspar and amphibole increased, and the contents of calcite and clay minerals remained stable. There were differences in mineral composition at the same depth. Quartz and potassium feldspar were the most abundant minerals in bare land L01, while amphibole and clay minerals were the least abundant. The plagioclase content was the largest in L02, the clay mineral content was the largest in L04, the amphibole content was the largest in L05, and the quartz and calcite contents were the lowest in L03. The soil microbial communities varied in different environments. The soil microbial community exhibited obvious commonness with increasing depth. The microbial community diversity at shallow depths was high, and its richness was low; the microbial community richness at deep depths was high, and its diversity was low. There were obvious differences in the microbial community diversity at the same depth. In bare land L01, the microbial community diversity was the largest, and richness was the lowest. The diversity and richness of the microbial community were low in L02, which was affected by fertilization, which indicated that fertilization had a great impact on the microbial community. Proteobacteria, Actinobacteria, and Acidobacteria were the dominant microorganisms in soil with increasing soil depth. The relative abundance of Nitrospirae increased with depth, whereas that of Chloroflexi exhibited the opposite pattern. In addition, as the depth increased, some microbes disappeared, while others emerged, such as Chlorobi and Chlamydiae. The dominant microflora of soil microorganisms differed at the same depth, such as Actinobacteria, Proteobacteria, and Acidobacteria in L01, L03, L04 and L05, while Actinobacteria, Proteobacteria and Firmicutes in L02 were dominant. The relative abundance of dominant flora in bare land L01 was the lowest, and the content of dominant flora was basically the same. The relative abundance of Firmicutes was very low in L01, L03, L04 and L05. Cyanobacteria do not exist in L02 and L03. Verrucomicrobia did not exist in L02 and L05. This result shows that there were obvious differences among the soil microbial species under different conditions. The phylogenetic analysis of microorganisms revealed that the same phylum exhibited a certain evolutionary relationship with depth. There was a relationship between species formation at different depths, such as Actinobacteria, Acidobacteria, and Proteobacteria.

Through comparative analysis of the soil microbial community and environmental factors, it was concluded that the environmental factors affecting the microbial community were different at different depths and at the same depths. The shallow microbial community was mainly affected by quartz and potassium feldspar, while the soil CO_2_ mainly affected the diversity of the deep soil microbial community. The microbial community of bare land L01 was mainly affected by potassium feldspar, L03 and L04 were mainly affected by calcite, and L02 was mainly affected by soil CO_2_. The dominant bacteria (Proteobacteria, Actinobacteria, and Acidobacteria) at different depths were positively correlated with carbon dioxide and amphibole but negatively correlated with quartz and K-feldspar. Plagioclase and K-feldspar had opposite effects on microbial flora. The results showed that CO_2_ and amphibole could improve the microbial community diversity at different depths, while quartz and K-feldspar could reduce the microbial diversity. At the same depth, amphibole and clay minerals were positively correlated with the dominant bacteria (Actinobacteria, Proteobacteria, and Acidobacteria), while Firmicutes was positively correlated with CO_2_. However, CO_2_ was negatively correlated with Firmicutes. The correlations between quartz and CO_2_ and the dominant bacteria were general. However, the subdominant bacteria Chloroflexi, Gemmatimonadetes, and Nitrospirae were negatively correlated with CO_2_. It is concluded that mineral components (amphibole and clay minerals) at the same depth were the main environmental factors affecting the diversity of soil microbial communities, while soil CO_2_ can affect the distribution of subdominant bacteria.

## Figures and Tables

**Table 1:** Sample information sheets (L01 and W01 were the same sampling points. To better compare different conditions, two numbers were adopted.).

Sample	W01	W02	W03	L01	L02	L03	L04	L05
Land use type	Bare	Bare	Bare	Bare	Peanut	Peach	Chestnut	Pine
Depth (cm)	0–15	30–45	60–75	0–15	0–15	0–15	0–15	0–15

**Table 2:** Results of mineral analysis by using X-ray diffraction of soil samples.

Samples	Quartz	K-Feldspar	Plagioclase	Calcite	Clay Minerals	Amphibole
L01 (W01)	27.4	29.9	33.7	1.3	7.1	0.5
L02	26.2	21.2	42.8	1.2	7.9	0.8
L03	19.2	27.7	42.3	1.1	8.2	1.5
L04	24.7	23.0	38.7	1.6	10.4	1.5
L05	26.5	19.6	40.6	1.2	10.0	2.0
W02	22.2	22.4	46.7	1.1	6.2	1.4
W03	21.0	26.0	42.2	1.4	7.2	2.2

**Table 3:** Soil bacterial diversity index based on 16S rDNA sequencing.

Sample	Shannon	Simpson	ACE	Chao1
W01	5.876	0.006062	997.07662	1013.0323
W02	5.860	0.005901	1031.5267	1028.75
W03	5.917	0.005626	1094.331	1096.1724
L01	6.020	0.004679	1280.969	1301.324
L02	4.606	0.060254	860.9973	858.8333
L03	5.990	0.005436	1354.22	1379.128
L04	5.989	0.005554	1325.689	1332.94
L05	5.667	0.017638	1316.49	1332.898
